# Unusual side effect from a luteinizing hormone-releasing hormone agonist, leuprorelin, in the treatment of prostate cancer: a case report

**DOI:** 10.1186/s13256-016-1110-5

**Published:** 2016-11-11

**Authors:** John I-Chiang Chang, Joseph Bucci

**Affiliations:** St George Hospital, Sydney, Australia

**Keywords:** Prostate cancer, Androgen deprivation therapy, Side effect, Lipodystrophy, Leuprorelin

## Abstract

**Background:**

The treatment options for high-risk prostate cancer are either radical prostatectomy or radiotherapy/brachytherapy depending on the patients’ prognosis. In older men with multiple comorbidities, radiotherapy with androgen deprivation therapy is an attractive option. Common side effects of androgen deprivation therapy include hot flushes, tiredness, increased risk of fractures, increased risk of metabolic disorders, coronary heart disease, and psychological effects. This case highlights the potential side effect of lipodystrophy secondary to leuprolide acetate injections. To the best of our knowledge, this is the first reported case of such an instance.

**Case presentation:**

In this case report, we describe a 70-year-old white man with prostate-specific antigen of 1.8 ng/mL, clinical stage T2bN0M0, Gleason 4+5=9 prostate cancer who developed an unusual side effect from leuprolide acetate as part of his androgen deprivation therapy. Approximately 2 months after the initial 3-monthly injection of leuprolide acetate (Eligard 22.5 mg) our patient developed abnormal lipid deposition particularly in his deltoid and abdominal region. His upper limb mobility gradually became compromised due to the size of these abnormal fat depositions. He had liposuction to correct this lipodystrophy and had a good functional outcome and cosmesis from the procedure.

**Conclusions:**

To the best of our knowledge, this is the first reported case of lipodystrophy secondary to leuprolide acetate injections. Leuprolide acetate in commonly used as one of the gonadotrophin-releasing hormone agonists and thus we should be mindful of the potential effect of producing lipodystrophy, especially in patients with cirrhosis, and to watch for any signs and symptoms as appropriate. The implication of this potential side effect poses difficult management strategies for such patients, and second-line alternatives such as chemotherapy may need to be considered.

## Background

The treatment of prostate cancer depends on the risk stratification and staging of the patient. Initial evaluation of a patient with suspected prostate cancer includes a detailed history, digital rectal examination, pre-treatment serum prostate-specific antigen (PSA), and Gleason score from the initial prostate biopsy. Imaging studies that are used include radionuclide bone scan and computed tomography (CT)/magnetic resonance imaging (MRI) of the abdomen and pelvis to look for nodal and metastatic involvement. With those findings patients are staged according to American Joint Committee on Cancer (AJCC)/Union for International Cancer Control (UICC) classifications [1, 2].

Based on their staging workup we can stratify patients into various classifications as suggested by the National Comprehensive Cancer Network (NCCN) [3]. Patients who are in the high-risk group are offered either radical prostatectomy with pelvic lymph node dissection or radiotherapy and androgen deprivation therapy with or without the addition of brachytherapy based on each patient’s life expectancy.

Eligard (leuprolide acetate also known as leuprorelin) is generally well tolerated, with the most common adverse event being flushing and injection site erythema [4]. It has not been reported to produce lipodystrophy. Lipodystrophy can either result from congenital disorders or acquired conditions. Lipodystrophy is most commonly associated with anti-retroviral medications in the treatment of human immunodeficiency virus (HIV) infections [5]. In this case report, we discuss an unreported side effect of lipodystrophy from leuprorelin injection as part of a patient’s androgen deprivation therapy.

## Case presentation

A 70-year-old white man presented with a history of lower urinary tract symptoms suggestive of prostatic enlargement. His background history included appendicectomy, diverticulitis, hypertension, and past heavy alcohol intake. He denied any significant family history. On examination, his prostate was found to be smooth and mildly enlarged. At this stage his PSA was measured at 1.8. Within 2 months it nearly doubled to 3.2 and a hard nodule was now felt on the right side of his prostate. He had a prostate biopsy under ultrasound guidance 3 months later and was found to have adenocarcinoma with Gleason score of 9 (5+4). Of the cores examined, 11 out of 17 were positive for adenocarcinoma. His prostate volume was 27 cc.

He underwent staging with a bone scan and CT of his chest, abdomen, and pelvis. There was no evidence of metastatic disease but there was evidence suggestive of liver cirrhosis on CT. After lengthy discussion with his urologist and radiation oncologist he proceeded with external beam radiotherapy with neoadjuvant and adjuvant androgen deprivation therapy. He received 76 Gy in 38 fractions using a conformal technique within 8 months of the prostate biopsy. He was also commenced on 3-monthly Eligard (leuprolide acetate also known as leuprorelin) subcutaneous injections (22.5 mg) over his lower abdomen for androgen deprivation.

**Fig. 1:**
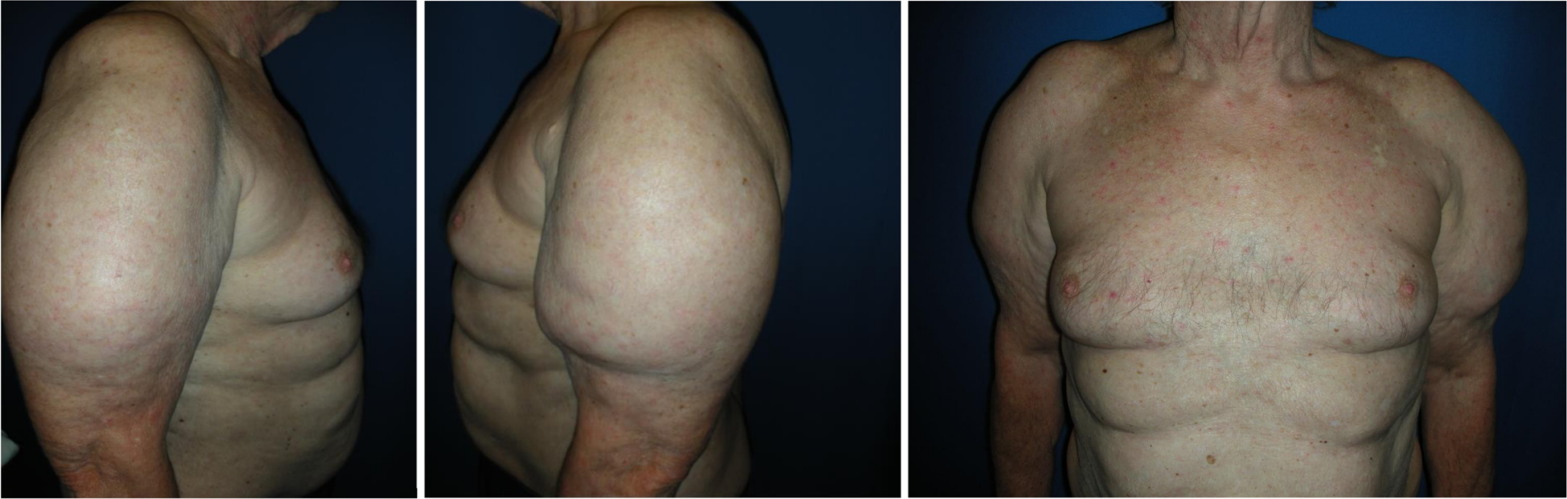
Before liposuction, 2 months post-initial leuprolide acetate injection

**Fig. 2:**
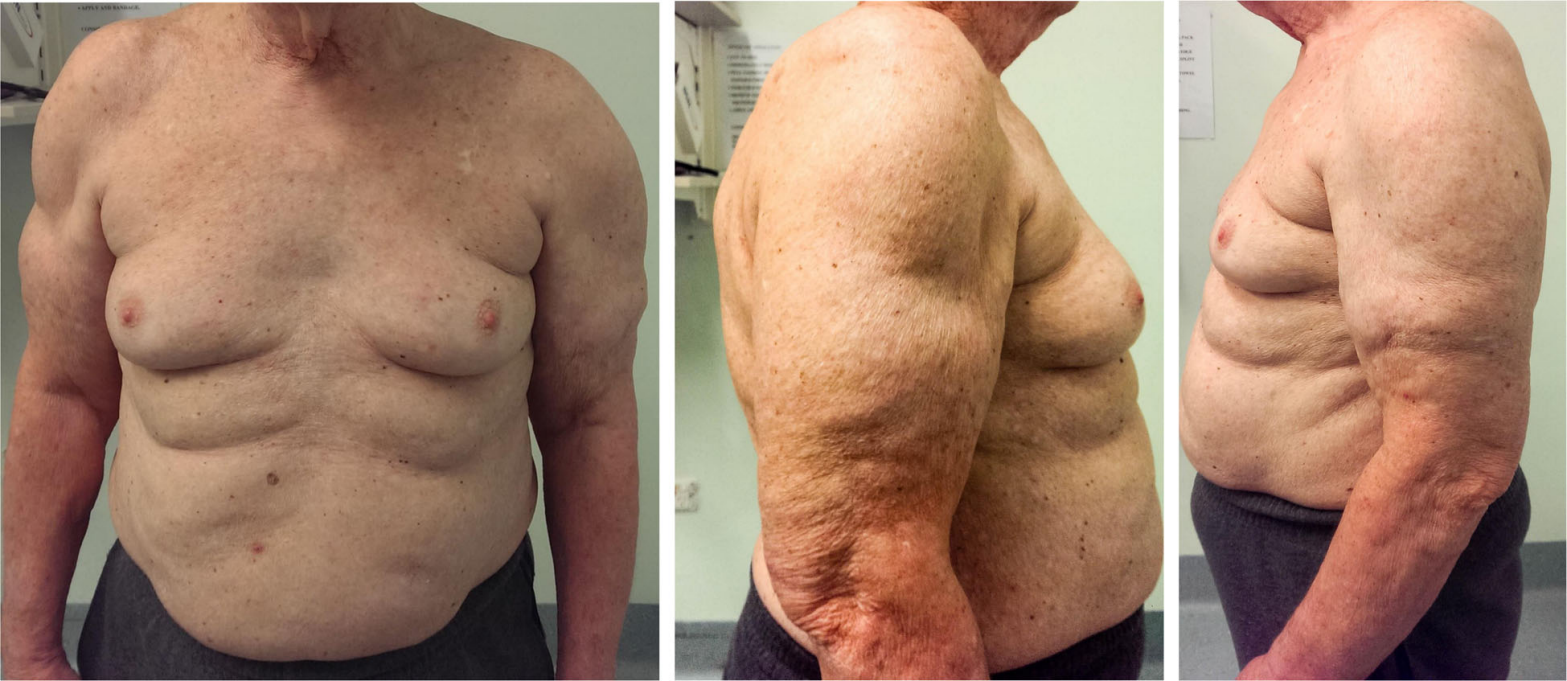
Six months post-liposuction

He started to note the development of abnormal swelling over his deltoids to the extent that it was interfering with his activities of daily living. The swelling was so large that he was unable to wear some shirts and he was unable to abduct and forward flex his shoulder joint which made reaching over his head to retrieve items from cupboards very difficult. He noticed these swellings over the 2 months post his first Eligard (leuprolide acetate also known as leuprorelin) injection (Fig. 1); however, this was not raised until one of his radiotherapy sessions. On examination he was noted to have a raised soft mass over his deltoids bilaterally measuring approximately 10 cm in diameter. His leuprolide injection was ceased. During the period of leuprolide treatment (9 months) he did not have any regimen change to his regular medications. His PSA and testosterone levels at the end of his radiotherapy were 0.03 ng/mL and 1 nmol/L respectively. Upon stopping the leuprolide injections he did note his hot flushes symptom had improved. A thorax CT was performed and confirmed these masses to be adipose tissue. He went to see a plastic surgeon who performed liposuction of the lipodystrophies on his deltoid regions. This had a good effect at 6 months postoperative follow up (Fig. 2).

On his most recent follow up, he unfortunately showed signs of clinical relapse with a rising PSA from 0.75 ng/mL and testosterone of 26.8 nmol/L (January 2014) to a PSA of 24 ng/mL and testosterone level of 34.1 nmol/L (September 2014) as well as evidence of bone metastases on a repeat bone scan. He was then recommenced on goserelin acetate 10.8 mg 3-monthly subcutaneous injection as a single agent for further treatment which has so far seemed successful.

## Discussion

The use of luteinizing hormone-releasing hormone (LHRH) agonists is not only limited to advance prostate cancer. They are commonly used in fertility treatments, endometriosis, precocious puberty, uterine fibroids, and hirsutism [6]. For fertility treatments, LHRH agonists are used in assisted reproduction technology to produce with a higher clinical pregnancy rate per cycle [7]. For treatment of pain from endometriosis, LHRH analogs produce a downregulation of the pituitary glands and thus result in ovarian suppression. This has been reported to be effective in upwards of 85 % of women [8]. Precocious puberty can also be treated by LHRH agonists and is known to be well tolerated and effective in suppressing luteinizing and follicle-stimulating hormones [9]. By the same principles as mentioned above, LHRH agonists can also be used in treating leiomyomas and hirsutism [10, 11]. The side effects for applications discussed above are the result of low estrogen level; symptoms such as hot flushes, tiredness, vagina dryness, and decreased libido are common. The most severe one is probably LHRH agonists’ effect on bone loss [12]. However there has been no mention of any lipodystrophy associated with LHRH agonists use in the literature.

It is interesting to note that lipodystrophy can also be seen in patients with HIV. The mechanism for this has not yet been proven but one theory suggests that it is related to HIV type 1 protease inhibitors and nucleoside reverse transcriptase inhibitors such as stavudine and zidovudine [13, 14].

Although the mechanism for the lipodystrophy in our patient is not clear, we postulate that due to the androgen surge that is associated with LHRH agonists such as leuprorelin [15], it created an environment where excess testosterone is available for the conversion to estrogen by aromatase. In a normal healthy liver there is usually little to no aromatase activity in the liver; however, in cirrhotic livers (such as in our patient from his previous heavy alcohol intake) it has been shown that there is increased aromatase activity and estrogen formation [16]. It is thought that this excess estrogen level is the cause for the abnormal fat deposition.

## Conclusions

In this case report we discuss an uncommon side effect from leuprolide acetate injections for the treatment of prostate cancer. To the best of our knowledge this is the first reported case of lipodystrophy secondary to leuprolide acetate injections. Leuprolide acetate is commonly used as one of the gonadotrophin-releasing hormone agonists and thus we should be mindful of the potential effect of producing lipodystrophy, especially in patients with cirrhosis, and to watch for any signs and symptoms as appropriate. The implication of this potential side effect poses difficult management strategies for such patients, and second-line alternatives such as chemotherapy may need to be considered.

## Abbreviations

AJCC: American Joint Committee on Cancer
CT: Computed tomography
HIV: Human immunodeficiency virus
LHRH: Luteinizing hormone-releasing hormone
MRI: Magnetic resonance imaging
NCCN: National Comprehensive Cancer Network
PSA: Prostate-specific antigen
UICC: Union for International Cancer Control
